# Risk of second primary malignancies in patients with chronic lymphocytic leukemia: a population-based study in the Netherlands, 1989-2019

**DOI:** 10.1038/s41408-023-00784-z

**Published:** 2023-01-13

**Authors:** Lina van der Straten, Mark-David Levin, Manette A. W. Dinnessen, Otto Visser, Eduardus F. M. Posthuma, Jeanette K. Doorduijn, Anton W. Langerak, Arnon P. Kater, Avinash G. Dinmohamed

**Affiliations:** 1 Department of Research and Development, Netherlands Comprehensive Cancer Organisation (IKNL), Utrecht, The Netherlands; 2 Department of Internal Medicine, Albert Schweitzer Hospital, Dordrecht, The Netherlands; 3 Department of Immunology, Erasmus MC, Rotterdam, The Netherlands; 4 Amsterdam UMC, University of Amsterdam, Department of Hematology, Cancer Center Amsterdam, Lymphoma and Myeloma Center Amsterdam, Amsterdam, The Netherlands; 5 Department of Registration, Netherlands Comprehensive Cancer Organisation (IKNL), Utrecht, The Netherlands; 6 Department of Internal Medicine, Reinier The Graaf Hospital, Delft, The Netherlands; 7 Department of Hematology, Leiden University Medical Center, Leiden, The Netherlands; 8 Erasmus MC Cancer Institute, Department of Hematology, University Medical Center Rotterdam, Rotterdam, The Netherlands; 9 Erasmus MC, Department of Public Health, University Medical Center Rotterdam, Rotterdam, The Netherlands; 10 Amsterdam UMC, Vrije Universiteit Amsterdam, Department of Hematology, Cancer Center Amsterdam, Amsterdam, The Netherlands

**Keywords:** Chronic lymphocytic leukaemia, Epidemiology, Preventive medicine

## Abstract

The longevity of patients with chronic lymphocytic leukemia (CLL) has improved progressively over the past decades, making it essential to understand long-term health outcomes, such as second primary malignancies (SPMs). Therefore, this nationwide, population-based study assessed the risk of SPM development in CLL patients diagnosed during 1989-2019 in the Netherlands compared to the expected number of malignancies in an age-, sex-, and period-matched group from the general Dutch population. In 24,815 CLL patients followed for 162,698.49 person-years, 4369 SPMs were diagnosed with a standardized incidence ratio (SIR) of 1.63 (95% confidence interval [CI] 1.59–1.68). This elevated risk was observed for solid (SIR, 1.67; 95% CI, 1.65–1.75) and hematological SPMs (SIR 1.42; 95% CI, 1.24–1.62). The highest risk for SPMs was noted beyond five years post-diagnosis (SIR, 1.70; 95% CI, 1.62–1.77), for male individuals (SIR, 1.70; 95% CI, 1.64–1.77), and patients aged 18–69 years (SIR, 1.92; 95% CI, 1.79–2.05). The risk of SPMs was higher in CLL patients who received anti-neoplastic therapy (SIR, 2.12; 95% CI, 1.96–2.28), as compared with those who did not (SIR, 1.58; 95% CI, 1.53–1.63). Routine surveillance activities and tailored interventions to counteract the increased morbidity and excess mortality associated with SPMs are essential for improving long-term outcomes in CLL patients.

## Introduction

Chronic lymphocytic leukemia (CLL) is the most frequently diagnosed leukemia among adults in the Western world, with an age-standardized incidence rate ranging from 3.8 to 5.0 per 100,000 person-years as of the 2000s [1–5]. The clinical behavior of CLL is heterogeneous, ranging from an indolent disease with a tendency to remain stable for many years without therapy to a more aggressive illness that rapidly relapses after initial treatment.

The past decades have witnessed significant progress in managing patients with CLL. More specifically, the most notable therapeutic breakthrough was the introduction of chemoimmunotherapy, which translated into improved outcomes for CLL patients at the population level [1, 6–8]. More recently, novel agents such as ibrutinib and venetoclax entered the therapeutic realm of CLL. Due to the availability of more efficacious therapies in the upfront and relapsed setting, the longevity of CLL patients improved progressively over time [1, 8]. Nevertheless, excess mortality among long-term CLL survivors persists and remains a threat in modern times [8, 9].

As the population of long-term CLL survivors is rapidly expanding, it is essential to understand long-term health outcomes. The development of second primary malignancies (SPMs) ―i.e., cancers diagnosed after CLL― may contribute to morbidity and offset the improved longevity of CLL patients. Therefore, awareness of the nature and magnitude of SPMs in CLL is essential for health-related planning and surveillance activities [10–14]. The relative scintilla of population-based studies in CLL has shown an increased risk of SPM development compared to the general population [15–18]. However, most of these studies have not investigated SPM development with long-term follow-up since the widespread use of chemoimmunotherapy in the 2010s and the most recent availability of novel targeted approaches. Also, most of these studies have included comparatively small cohorts with a short follow-up time. Therefore, to complement and extend the currently sparse literature on SPM development in CLL, this nationwide, population-based study aimed to assess temporal trends in SPM development —compared with an age-, sex- and period-matched group of the general population— in various subgroups of CLL patients in the Netherlands during a 30-year period that takes into account the treatment advances of CLL during that period.

## Patients and methods

### The Netherlands Cancer Registry

Established in 1989, the Netherlands Cancer Registry (NCR), which is maintained and hosted by the Netherlands Comprehensive Cancer Organisation (IKNL), has an overall coverage of at least 95% of all newly diagnosed malignancies in the Netherlands [19]. The NCR relies on comprehensive case notification via the Nationwide Network and Registry of Histopathology and Cytopathology and the National Registry of Hospital Discharges (i.e., inpatient and outpatient discharges). Basic information on dates of birth and diagnosis, sex, primary therapy, and disease stage, topography, and morphology of all newly diagnosed malignancies are routinely ascertained in the NCR by trained registrars of IKNL through retrospective review of medical records. Topography and morphology are coded as per the International Classification of Diseases for Oncology (ICD-O) [20]. Information on vital status (i.e., alive, dead, or emigration) is obtained through an annual linkage with the Nationwide Population Registries Network that holds this information for all residents in the Netherlands.

### Study population

All patients diagnosed with CLL between January 1, 1989, and December 31, 2019, were selected from the NCR using the ICD-O morphology code 9823. Patients diagnosed at autopsy (*n* = 71) were excluded from the analysis. Through cross-linkage with the NCR, SPMs diagnosed between 1989 and 2019 were identified. The ICD-O morphology and topography codes used to categorize specific SPM groupings are depicted in Supplemental Table 1 [20]. Basal cell carcinomas of the skin were excluded from the analysis because these malignancies were not standardly ascertained throughout the study period. Also, diffuse large B-cell lymphomas (DLBCL) and Hodgkin lymphomas were excluded since these lymphomas might have been misclassified as SPMs when they may be transformations of CLL (i.e., Richter’s syndrome). Finally, synchronous SPMs diagnosed within six months after CLL diagnosis were excluded to minimize surveillance bias since these SPMs might be incidental findings rather than true SPMs. Patients with multiple and metachronous SPMs were counted only once in the analysis for all sites and all solid and hematological SPMs combined. However, these subsequent cancers contributed to the cancer site-specific analysis regardless of whether it was preceded by a malignancy from another site [21].

According to the Central Committee on Research involving Human Subjects (CCMO), this type of observational, non-interventional study does not require approval from an ethics committee in the Netherlands. The Privacy Review Board of the NCR approved the use of anonymous data for this study.

### Primary therapy

The NCR generally ascertains information on primary therapy initiated within one-year postdiagnosis. For the overall analysis (i.e., 1989–2019), primary therapy was grouped into (i) no anti-neoplastic therapy, including a watch-and-wait approach, and (ii) anti-neoplastic therapy. The latter group was subdivided into chemotherapy alone and chemoimmunotherapy. Of note, the NCR ascertains the use of rituximab as of January 1, 2007. However, the use of rituximab before 2007 in the frontline management of CLL is presumed to be neglectable, as rituximab was initially introduced around 2010 for previously untreated CLL patients in combination with fludarabine and cyclophosphamide [22].

As of January 1, 2014, information on the exact therapeutic regimens was registered in the NCR. These regimens were categorized as fludarabine, cyclophosphamide and rituximab (FCR), bendamustine and rituximab (BR), rituximab, cyclophosphamide, vincristine and prednisone (R-CVP), rituximab or obinutuzumab with chlorambucil (R- or O-Clb), chlorambucil monotherapy, ibrutinib, venetoclax, and other less frequently applied modalities. Of note, ibrutinib was reimbursed in the Netherlands as of late 2014 and venetoclax as of 2017 for previously untreated CLL patients harboring *TP53* aberrations [23–26].

### Statistical analyses

Person-years at risk were calculated from the date of CLL diagnosis until SPM diagnosis, death, or end of follow-up (December 31, 2019), whichever occurred first. The risk time ended at the diagnosis of the first SPM of interest in the case of multiple SPMs within one patient. Standardized incidence ratios (SIRs) were computed as the ratio of observed SPMs to expected SPMs from the general population. The expected number of malignancies was based on age-, sex-, calendar, and site-specific cancer-incidence rates in the Dutch population, which were multiplied by the corresponding person-years at risk. The absolute excess risk (AER) represents the additional incidence of SPMs measured beyond the background incidence of SPMs found in the Dutch general population. The AER was calculated as the expected number of SPMs subtracted by the observed number of SPMs, divided by the person-years at risk and multiplied by 10,000, resulting in an AER per 10,000 person-years [27, 28]. Poisson distribution for the number of observed SPMs was assumed to calculate the 95% confidence intervals (CIs) for the SIR and AER. Unless otherwise stated, the SIRs and AERs were presented overall and according to age category (18–69 and ≥70 years), sex, the latency period for SPM development defined as the years from CLL diagnosis until SPM development (0.5–5 and ≥5 years), calendar period (1989–1995, 1996–2002, 2003–2009, and 2010–2019) and the receipt of anti-neoplastic therapy (no *versus* yes). The calendar periods were used as a proxy for the evolution of therapeutic modalities over time. The criteria of non-overlapping CIs were used to show statistically significant differences between subgroups [27].

The cumulative incidence of SPMs was evaluated, with death treated as a competing risk. The expected cumulative incidence in the general population was derived from the expected cancer incidence rates and expected overall mortality rates in the Dutch general population. The cumulative incidence was estimated for all sites, all solid and hematological SPMs, and individual SPM subtypes (Supplemental Table 1).

A multivariable analysis was performed using the Fine and Grey method to analyze the effect of age (18–59, 60–69, 70–79, and ≥80 years), sex, calendar period (1989–1995, 1996–2002, 2003–2009, and 2010–2019), and receipt of anti-neoplastic therapy on the cumulative incidence of SPMs [29].

All statistical analyses were performed with STATA Statistical Software version 17.0 (StataCorp, College Station, TX) and SAS version 9.4 (SAS Institute Inc, Cary, North Carolina, USA).

## Results

Our analytic cohort included 24,815 CLL patients (61% males; median age 69 years; interquartile age range [IQR], 61–67 years) diagnosed in the Netherlands between 1989 and 2019. The baseline and primary treatment characteristics are presented in Table 1 according to the calendar period of diagnosis. Overall, the median follow-up period was 6.2 years (IQR, 3.2–10.6 years), with 28% of the patients being followed for at least ten years. This overall follow-up period resulted in a total follow-up of 162,698.49 person-years.

**Table 1:** Patient and primary therapy characteristics according to the calendar period of diagnosis.

	1989–1995		1996–2002		2003–2009		2010–2019		Total	
Characteristics	No.	(%)	No.	(%)	No.	(%)	No.	(%)	No.	(%)
**Number of patients**	3519		4356		6190		10,750		24,815	
ASR*	3.23		3.64		4.54		4.53		4.04	
**Sex**										
Male	2047	(58)	2602	(60)	3759	(61)	6693	(62)	15,101	(61)
Female	1472	(42)	1754	(40)	2431	(39)	4057	(38)	9714	(39)
**Age**										
Median (IQR)	70 (62–78)		69 (61–77)		69 (60–77)		69 (62–77)		69 (61–77)	
18–59	652	(19)	979	(22)	1412	(23)	2056	(19)	5099	(21)
60–69	1018	(29)	1213	(28)	1828	(30)	3328	(31)	7387	(30)
70–79	1158	(33)	1457	(33)	1898	(31)	3462	(32)	7975	(32)
≥80	691	(20)	707	(16)	1052	(17)	1904	(18)	4354	(18)
**Follow-up years**										
Median (IQR)	5.6 (2.1–11.2)		7.4 (2.9–15.2)		9.7 (4.2–13.9)		5.3 (3.2–7.8)		6.2 (3.2–10.6)	
**Latency period**^**#**^										
0.5–5	1630	(46)	1643	(38)	1802	(29)	5068	(38)	10,143	(41)
6–10	857	(24)	1007	(23)	1357	(22)	4533	(23)	7754	(31)
11–15	428	(12)	593	(14)	1952	(32)	1149	(14)	4122	(17)
16–20	247	(7)	528	(12)	1079	(17)	0	(12)	1854	(8)
21–24	136	(4)	533	(12)	0	(0)	0	(12)	669	(3)
≥25	211	(6)	52	(1)	0	(0)	0	(1)	273	(1)
**Anti-neoplastic therapy**^†^										
Yes	892	(26)	993	(23)	967	(16)	1217	(11)	4069	(16)
No/unknown	2627	(74)	3363	(77)	5223	(84)	9533	(89)	20,746	(84)

*ASR* age-standardized incidence rate, *IQR* interquartile range, *No* number.

* ASRs are age-adjusted according to the European standard population and expressed per 100,000 person-years.

^#^ The latency period is defined as the time from CLL diagnosis until SPM development, death, emigration or last follow-up.

^†^ Anti-neoplastic therapy initiated as primary therapy within one year after the CLL diagnosis.

In the overall series, most patients did not receive anti-neoplastic therapy, including a watch-and-wait approach, within one year post-diagnosis (84%; Table 1). The use of anti-neoplastic therapy decreased with each successive calendar period, following a broader institution of a watch-and-wait approach. The gradual increase in the age-standardized incidence rate between 1989–1995 (3.23 per 100,000 person-years) to 2003–2009 (4.54 per 100,000 person-years) was followed by a stabilization during 2010–2019 (4.53 per 100,000 person-years). This finding may suggest that the initial increase might be attributed to higher detection of early-stage CLL and consequently might explain the higher proportion of watch-and-wait approaches over time (Table 1). Of the patients receiving anti-neoplastic therapy as of 2007, 595 (37%) patients received chemotherapy and 893 (55%) chemoimmunotherapy. For patients diagnosed as of 2014 and receiving anti-neoplastic treatment, the majority of the patients received FCR (27%) followed by R- or O-Clb (26%), chlorambucil monotherapy (10%), R-CVP (9%), BR (9%), other, less frequently applied treatments (9%), venetoclax-based (5%), and ibrutinib-based treatment (4%).

### Risk of second primary malignancies as compared to the general population

During the follow-up period, 4,700 SPMs were diagnosed in 4,369 CLL patients. SPMs were diagnosed after a median follow-up period of 4.2 years post-CLL diagnosis (IQR, 1.6–7.9 years) and at a median age of 74 years (IQR, 68–82 years). The cumulative incidence of SPM development was 29.45% (95% CI, 28.66%-30.26%), 50.84% (95% CI, 49.20%-52.52%) and 69.18% (95% CI, 63.50%-74.71%) at 10, 20, and 30 years respectively (Table 1). The cumulative incidence of all SPM subtypes is depicted in Supplemental Fig. 1.

Overall, the risk of developing an SPM was significantly higher among CLL patients compared to the general population (SIR, 1.63; 95% CI, 1.59–1.68), resulting in 125.06 excess malignancies per 10,000 person-years (Table 2). This increased risk of SPM development was observed for both solid (SIR, 1.67; 95% CI, 1.65–1.75) and hematological SPMs (SIR, 1.42; 95% CI, 1.24–1.62; Table 2). The risk was more than two-fold increased among CLL patients compared to the general population for squamous cell carcinomas of the skin (SIR, 4.82; 95% CI, 4.57–5.07), acute myeloid leukemia (AML; SIR, 2.75; 95% CI, 2.08–3.58), melanomas of the skin (SIR, 2.74; 95% CI, 2.43–3.08), soft-tissue sarcomas (SIR, 2.39; 95% CI, 1.70–3.27), and thyroid cancers (SIR, 2.12; 95% CI, 1.26–3.35; Table 2). Although relative risks were doubled, the absolute risk for AML, soft-tissue sarcomas and thyroid cancers remained comparatively low—reflected by a 30-year cumulative incidence of 1.90%, 1.12%, and 0.17%, respectively—owing to the low background risk for these malignancies in the general population (Table 2 and Supplemental Fig. 1).

**Table 2:** Standardized incidence ratios, absolute excess risk, and 30-year cumulative incidence of second primary malignancies in patients with chronic lymphocytic leukemia.

Type of SPM	No. of patients	Standardized incidence ratio (95%CI)*		Absolute excess risk per 10,000 person-years	30-year cumulative incidence (95%CI)	
**All sites**	4369	**1.63**	**(1.59–1.68)**	125.06	69.18	(63.50–74.71)
**Any solid cancer**	4224	**1.67**	**(1.65–1.75)**	122.51	67.69	(61.80–73.46)
**Oral cavity or pharynx**	54	1.11	(0.83–1.45	0.36	0.86	(0.56–1.30)
**Gastrointestinal tract**						
Esophagus	67	0.99	(0.77–1.26)	−0.03	1.08	(0.66–1.75)
Stomach	87	1.20	(0.96–1.48)	0.95	1.35	(0.91–1.99)
Colon and rectum	533	**1.21**	**(1.11–1.31)**	6.13	11.93	(8.87–15.96)
Pancreas	84	1.13	(0.90–1.40)	0.65	3.38	(1.51–7.46)
**Lower respiratory system**						
Larynx	30	1.21	(0.82–1.73)	0.34	0.70	(0.38–1.28)
Lung or bronchus	573	**1.36**	**(1.25–1.47)**	9.98	13.29	(9.51–18.41)
**Skin**						
Melanoma	278	**2.74**	**(2.43–3.08)**	11.72	4.30	(3.38–5.45)
Squamous cell	1,487	**4.82**	**(4.57–5.07)**	80.31	34.37	(28.87–40.58)
**Breast**	259	1.06	(0.94–1.20)	1.05	5.20	(3.62–7.43)
**Female genital organ**						
Endometrium	43	1.04	(0.75–1.40)	0.11	0.65	(0.45–0.96)
Ovary	28	0.97	(0.64–1.40)	−0.06	0.41	(0.25–0.66)
**Male genital organ**						
Prostate	476	1.10	(1.00–1.20)	2.80	12.31	(6.28–23.34)
**Urinary tract**						
Urinary bladder or renal pelvis	152	1.16	(0.98–1.36)	1.36	2.20	(1.62–2.99)
Kidney	120	**1.73**	**(1.44–2.07)**	3.36	3.20	(2.23–4.59)
**Brain**	36	1.35	(0.95–1.87)	0.62	1.19	(0.61–2.34)
**Thyroid gland**	18	**2.12**	**(1.26–3.35)**	0.63	0.17	(0.11–0.28)
**Soft-tissue sarcoma**	39	**2.39**	**(1.70–3.27)**	1.50	1.12	(0.53–2.32)
**Primary site unknown**	129	**1.67**	**(1.39–1.98)**	3.40	2.65	(1.82–3.87)
**Blood, bone marrow, or lymphatic system**						
Any hematological cancer	224	**1.42**	**(1.24–1.62)**	4.41	5.07	(3.65–7.04)
Non-Hodgkin lymphoma	59	**1.37**	**(1.04–1.76)**	1.04	0.80	(0.56–1.12)
Multiple myeloma	40	1.03	(0.74–1.40)	0.08	0.72	(0.45–1.15)
Acute myeloid leukemia	56	**2.75**	**(2.08–3.58)**	2.35	1.90	(0.91–3.95)
Myeloproliferative neoplasm	17	0.66	(0.39–1.06)	−0.57	0.57	(0.22–1.47)
Myelodysplastic syndromes	35	1.34	(0.94–1.87)	0.59	0.96	(0.44–2.07)

*CI* confidence interval, *No*. number.

*The listed cancers are those of which at least 10 cases were observed in the cohort. Statistically significant standardized incidence rates are presented in bold in the table.

Squamous cell carcinomas of the skin contributed most to the overall excess risk (AER, 80.31/10,000 person-years), representing 64% of the total excess risk, followed by melanomas of the skin (9%; AER, 11.72/10,000 person-years), lung and bronchus cancer (8%; AER, 9.98/10,000 person-years), colon and rectum cancer (5%; AER, 6.13/10,000 person-years), and kidney cancer (3%; AER, 3.36/10,000 person-years; Table 2).

### Relative and absolute excess risk according to sex

**Fig. 1:**
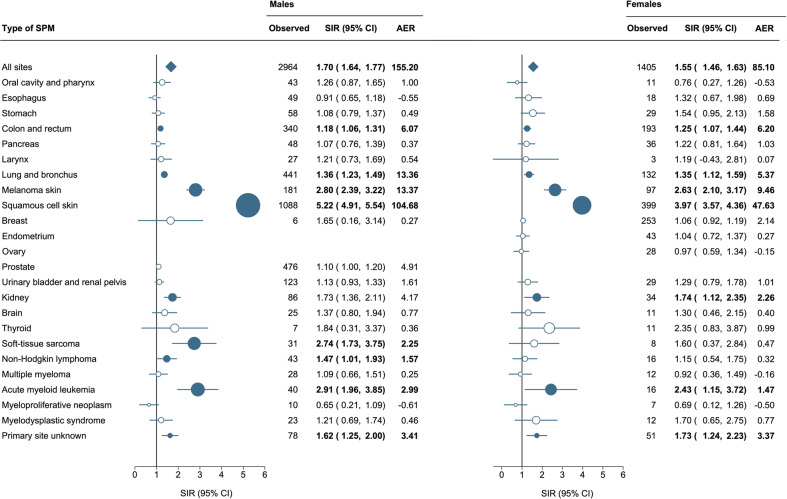
Risk of second primary malignancies among patients with chronic lymphocytic leukemia according to sex. Statistically significant standardized incidence ratios are presented in bold in the table and as solid dark blue dots in the forest plot and are scaled according to their magnitude. Abbreviations: AER, absolute excess risk; CI, confidence interval; and SIR, standardized incidence ratio.

The SIRs for any SPM were statistically higher for males (SIR, 1.70; 95% CI, 1.64–1.77) than for females (SIR, 1.55; 95% CI, 1.46–1.63). Also, the AER was nearly 2-fold higher for males than females (155.20 *versus* 85.10 per 10,000 person-years). The spectrum of SPMs for male and female CLL patients is depicted in Fig. 1. Generally, the spectrum of SPMs was comparable across the sexes except for the risk of soft-tissue sarcomas (SIR, 2.74; 95% CI, 1.73–3.75) and non-Hodgkin lymphomas excluding DLBCLs (SIR, 1.47; 95% CI, 1.01–1.93), which was only heightened in males. Although the SIRs for squamous cell carcinoma of the skin were increased for both sexes, the SIRs for males (SIR, 5.22; 95% CI, 4.91–5.54) were significantly higher than for females (SIR, 3.97; 95% CI, 3.57–4.36).

### Relative and absolute excess risk according to age

Overall, the SIRs were the highest for patients aged 18–59 years (SIR, 1.92; 95% CI, 1.79–2.06) compared with those aged 60–69 years (SIR, 1.60; 95% CI, 1.52–1.68), 70–79 years (SIR, 1.57; 95% CI, 1.49–1.65) and ≥80 years (SIR, 1.57; 95% CI, 1.44–1.71; Supplemental Fig. 2A). However, the absolute risk increased with advancing age, ultimately reaching 155.24 excess malignancies per 10,000 person-years in patients aged ≥80 years (Supplemental Fig. 2B). This inverse correlation is probably due to a lower background risk for developing SPMs in younger individuals.

**Fig. 2:**
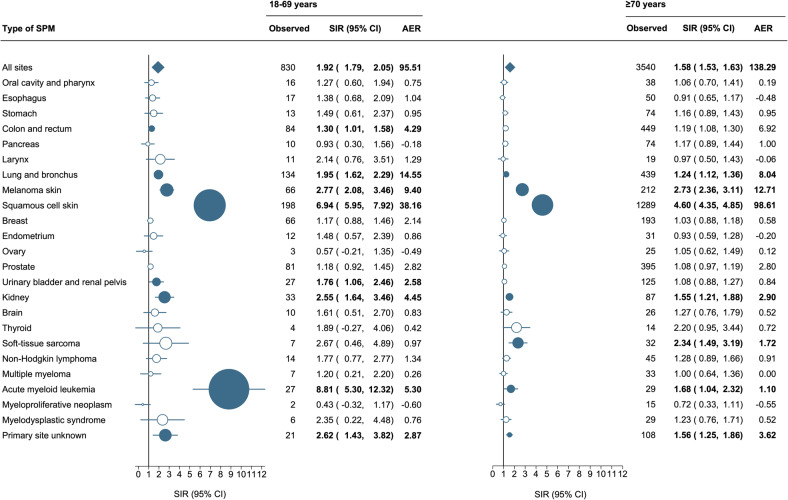
Risk of second primary malignancies among patients with chronic lymphocytic leukemia to age. Statistically significant standardized incidence ratios and absolute excess risk are presented in bold in the table and as solid dark blue dots in the forest plot and are scaled according to their magnitude. AER absolute excess risk, CI confidence interval, SIR standardized incidence ratio.

For the site-specific analysis, the SIRs and AERs were reported for patients aged 18–69 years and ≥70 years (Fig. 2). The risk of colon and rectum carcinomas (SIR, 1.30; 95% CI, 1.01–1.58) and urinary bladder and renal pelvis carcinomas (SIR, 1.76; 95% CI, 1.06–2.46) were only elevated in individuals aged 18–69 years, whereas the risk of soft-tissue sarcomas (SIR, 2.34; 95% CI, 1.49–3.19) was only elevated in ≥70 years old CLL patients. Of note, the risk of AML was more than five times higher in individuals aged 18–69 years (SIR, 8.81; 95% CI, 5.30–12.32) compared to those ≥70 years (SIR, 1.68; 95% CI, 1.04–2.32), probably owing to higher exposure to fludarabine-based therapies.

### Trends of second primary malignancies according to the calendar period of diagnosis

The risk of developing any SPM remained elevated compared to the general population during all the studied calendar periods (Supplemental Table 2). Overall, the SIRs and the AERs were comparable across all the calendar periods. The SIRs for solid cancers were significantly elevated and remained relatively stable over time. On the contrary, a heightened risk of hematological cancers only emerged in the last two calendar periods (SIR_2003–2009_, 1.55; 95% CI, 1.24–1.91 and SIR_2010–2019_, 1.63; 95% CI, 1.27–2.05). This elevation was noticeable from 2003 onwards and was attributed to a significantly higher risk for AML and myelodysplastic syndromes (MDS) in the latter period. Also, the risk of thyroid cancer was significantly higher in the most recent calendar period only. Conversely, the risk of lung and bronchus cancer, kidney cancer, soft-tissue sarcomas, and cancers with an unknown origin was higher among CLL patients in the earlier studied periods and lost significance in the latter calendar period. Of note, the risk of squamous cell carcinomas of the skin and melanomas of the skin remained significantly elevated throughout the entire study period.

### Trends of second primary malignancies according to the latency period

Overall, the SIRs for any SPMs remained significantly higher throughout different latency periods among CLL patients than in the general population, even after 21–30 years post-diagnosis (SIR, 1.86; 95% CI, 1.47–2.34; Supplemental Fig. 3A). Also, the SIRs remained comparatively stable throughout different latency periods. However, the absolute risk was significantly lower for patients with a latency time of 0.5–5 years (AER, 109.00; 95% CI, 101.77–116.90) compared to the overall excess risk (AER, 125.06; 95% CI, 119.25–131.23). The absolute risk increased steadily over the latency time, reaching 212.04 excess malignancies per 10,000 person-years after more than 20 years of follow-up (Supplemental Fig. 3B).

**Fig. 3:**
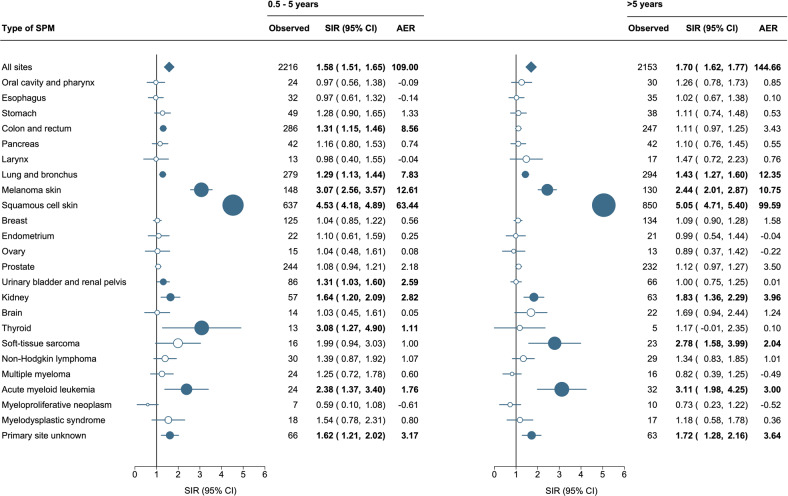
Risk of second primary malignancies among patients with chronic lymphocytic leukemia according to the latency time. Statistically significant standardized incidence ratios and absolute excess risk are presented in bold in the table and as solid dark blue dots in the forest plot and are scaled according to their magnitude. AER absolute excess risk per 10,000 person-years, CI confidence interval, SIR standardized incidence ratio.

For the site-specific analysis, the SIRs and AERs were reported for a latency period of 0.5–5 years and ≥5 years (Fig. 3). The spectrum of SPM subtypes varied across the latency periods. More specifically, the risk of colon and rectum cancers (SIR, 1.31; 95% CI, 1.15–1.46), urinary bladder and renal pelvis cancers (SIR, 1.31; 95% CI, 1.03–1.60), and thyroid cancers (SIR, 3.08; 95% CI, 1.27–4.90) was significantly higher among patients with CLL that developed an SPM within 0.5–5 years, as compared with the general population. On the other hand, the risk of developing a soft-tissue sarcoma (SIR, 2.78; 95% CI, 1.58–3.99) was only elevated after a latency time of ≥5 years.

### Effect of primary therapy on standardized incidence ratios

**Fig. 4:**
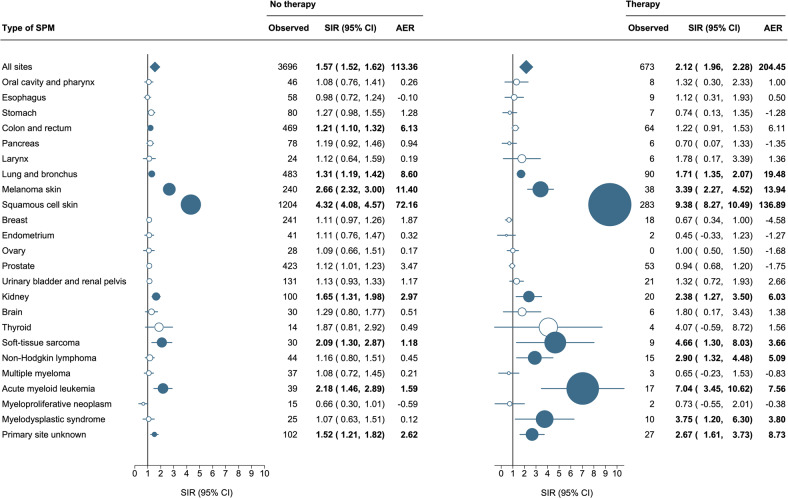
Risk of second primary malignancies among patients with chronic lymphocytic leukemia according to the receipt of anti-neoplastic therapy. Statistically significant standardized incidence ratios and absolute excess risk are presented in bold in the table and as solid dark blue dots in the forest plot and are scaled according to their magnitude. AER absolute excess risk, CI confidence interval, SIR standardized incidence ratio.

The SIRs and AERs were significantly higher in the patients who received anti-neoplastic therapy within one year post-diagnosis (SIR, 2.12; 95% CI, 1.96–2.28) as compared to those who did not (SIR, 1.57; 95% CI, 1.52–1.62), attributing to 204.45 and 113.36 excess cases per 10,000 person-years, respectively (Fig. 4). The risk of developing any hematological cancer (SIR, 3.11; 95% CI, 2.34–4.03), AML (SIR, 7.04; 95% CI, 3.45–10.62), MDS (SIR, 3.75; 95% CI, 1.20–6.30), and non-Hodgkin lymphomas (SIR, 2.90; 95% CI, 1.32–4.48) was significantly higher among patients who received anti-neoplastic therapy as compared to those who did not. Also, the risk of squamous cell carcinomas of the skin was significantly higher and more than 2-fold higher among CLL patients receiving treatment (SIR, 9.38; 95% CI, 8.27–10.49) as compared with those that did not (SIR, 4.32; 95% CI, 4.08–4.57). Of note, the risk of colon and rectum carcinomas was only elevated in untreated patients (SIR, 1.21; 95% CI, 1.10–1.32).

The SIRs for patients treated with chemotherapy (SIR, 2.16; 95% CI, 1.98–2.35) and chemoimmunotherapy (SIR, 2.09; 95% CI, 1.72–2.52) were comparable. As for patients diagnosed with CLL between 2014 and 2019, the spectrum of SPM development per treatment category is listed in Supplemental Table S3. Of note, these data do not show the SIRs and AERs due to the comparatively low number of SPMs.

### Multivariable effects on second primary malignancy development within the cohort

Next, we fitted a multivariable competing risk model for evaluating the effect of baseline patient characteristics and primary therapy on the risk of developing an SPM within our cohort (Table 3). The sHR of developing an SPM was higher in males (sHR, 1.45; 95% CI, 1.37–1.54) and in patients who received anti-neoplastic therapy within one-year postdiagnosis (sHR, 1.19; 95% CI, 1.09–1.27). The sHR of developing an SPM was higher in individuals aged 60–69 (sHR, 1.39; 95% CI, 1.29–1.50) and 70–79 years (sHR, 1.40; 95% CI, 1.29–1.51) as compared with those aged 18–59 years. Conversely, elderly patients ≥80 years had a significantly lower risk (sHR, 0.88; 95% CI, 0.79–0.98), probably caused by a higher probability of death in very elderly individuals. The cumulative incidence of SPMs increased by calendar period and was statistically higher in 1996–2002 (sHR, 1.11; 95%CI, 1.01–1.22), in 2003–2009 (sHR, 1.31; 95% CI, 1.20–1.43) and in 2010–2019 (sHR, 1.34; 95% CI, 1.22–1.46) as compared to the calendar period of 1989–1995.

**Table 3:** Univariable and multivariable competing risk model for the cumulative incidence of SPM development, with death considered as a competing event.

	Univariable			Multivariable		
	sHR (95% CI)		*P*-value	sHR (95% CI)		*P* value
**Sex**						
Female	(ref)			(ref)		
Male	1.48	1.40–1.57	<0.001	1.45	1.37–1.54	<0.001
**Age category (years)**						
18–59	(ref)			(ref)		
60–69	1.39	1.29–1.50	<0.001	1.39	1.29–1.50	<0.001
70–79	1.37	1.27–1.48	<0.001	1.40	1.29–1.51	<0.001
≥80	0.85	0.76–0.94	0.002	0.88	0.79–0.98	0.020
**Calendar period**						
1989–1995	(ref)					
1996–2002	1.12	1.02–1.23	0.015	1.11	1.01–1.22	0.030
2003–2009	1.34	1.23–1.46	<0.001	1.31	1.20–1.43	<0.001
2010–2019	1.38	1.26–1.51	<0.001	1.34	1.22–1.46	<0.001
**Receipt of anti-neoplastic therapy**						
No/unknown	(ref)			(ref)		
Yes	1.21	1.11–1.31	<0.001	1.19	1.09–1.27	<0.001

*CI* confidence interval, *ref* reference, *sHR* subhazard ratio.

## Discussion

In this large, nationwide, population-based study with long-term follow-up, we observed that CLL patients have a 63% higher risk of developing any SPM than an age-, sex, and calendar period-matched group from the general Dutch population. This risk for developing solid and hematological SPMs was 67% and 42% higher, respectively. This finding aligns with previously reported estimates from an Australian, Danish and U.S. study [15, 16, 18]. The spectrum of SPMs was also broadly comparable and mainly consisted of squamous cell carcinomas of the skin, melanomas of the skin, lung and bronchus cancer, colon and rectum cancer, soft-tissue sarcomas, AML, and thyroid cancer [14, 15, 18, 30]. The study from the U.S., which was based on data from the Surveillance, Epidemiology, and End Results (SEER) Program, reported the highest risk for Kaposi sarcomas with a SIR of 3.82 (95% CI, 2.19–6.21). Since the number of patients diagnosed with Kaposi sarcoma within our cohort was less than 10, we did not incorporate it in our analysis. Differences in HIV incidence might cause a lower incidence of Kaposi sarcoma in the Netherlands than in the U.S. Indeed, the incidence of HIV in 2020 was 9.2 and 2.3 per 100,000 person-years in the U.S. and the Netherlands, respectively [31, 32]. Also, as shown in the U.S. study, we could not objectify the decreased risk of hepatobiliary, breast, and uterine cancers [15]. Since there is currently no clear pathophysiological explanation for these associations, this warrants further validation in forthcoming studies.

The SEER-based analysis reported the highest incidence of SPMs to be diagnosed between 2 and 6 months after the CLL diagnosis; thereafter, the incidence of SPMs was lower and remained comparatively stable [15]. Notably, we observed that the magnitude of the SIRs remained stable across the different latency periods. However, a significantly lower AER for SPMs was observed within 0.5–5 years after the CLL diagnosis. This observation is probably related to excluding synchronous malignancies diagnosed within six months after the CLL diagnosis to minimize surveillance bias due to heightened medical care. Also, this finding highlights that a longer follow-up time is needed to capture the effect of impaired immune surveillance, environmental exposures, and chemotherapeutics on SPM development [33].

In line with previous studies, we observed that the SIRs were the highest in younger individuals while the AER increased with advancing age, the latter being attributed to a greater background incidence of SPMs in the general population. On the other hand, we observed an increased cumulative incidence of SPMs in CLL patients aged 60–79 years. This finding suggests that the SPM risk progressively increases with age, which might be explained by the improved longevity and the accumulation of risk factors [15, 34, 35].

The SIRs and AERs were higher in male CLL patients than in female CLL patients. The greater SPM risk in males is attributed to a higher risk of squamous cell carcinomas of the skin, which might be explained by a higher likelihood of males working outdoors with concomitant higher UV exposure [36, 37].

We noted an increasing cumulative incidence of SPMs in the most recent calendar periods, driven mainly by a higher number of hematological cancers rather than an increase in solid cancers, which actually remained relatively stable over time. Among the hematological SPMs, the risk of AML and MDS increased as of the 2000s, likely due to a higher application of fludarabine-based therapies from that time onwards. Indeed, this trend was previously described in the SEER-based analysis and in a single-center study in which patients were uniformly treated with FCR [12, 15]. In addition, the German CLL study group (GCLLSG) registry study demonstrated a higher-than-expected incidence of hematological SPMs (SIR, 3.64; 95% CI, 1.66–6.90) in treated *versus* untreated CLL patients within prospective studies as compared with the German general population [38]. An excess of skin malignancies, including squamous cell carcinomas and melanomas, seems to characterize CLL patients, and the burden is known to increase with therapy [39–41]. Lastly, apart from surveillance bias, it is proposed that CLL-related therapy and the immune dysfunctional nature of CLL might enhance the effect of common carcinogens, such as UV exposure and smoking, in increasing the probability of skin and respiratory cancers [14, 33].

The advent of targeted treatment approaches has transformed CLL management, which, in turn, has improved patient survival. As for the latter, long-term health risks, including SPMs, are becoming increasingly important because the improved longevity of CLL patients may be offset by these unwanted risks [42]. In patients treated with Bruton’s tyrosine kinase inhibitors, the risk and the spectrum of SPMs were similar to that reported following chemotherapy or chemoimmunotherapy [11, 43]. In our cohort we could not calculate the SIRs for patients receiving novel approaches due to the detection of only two SPMs and a short follow-up time in the last calendar period (2014–2019) in which these agents have become available in the Netherlands [44]. When using the calendar period as a proxy for the evolution of treatment over time, the risk and the spectrum of SPMs were comparable for the 2003–2009 and 2010–2019 periods, suggesting that both the introduction of chemoimmunotherapy and, in part, targeted therapies did not dramatically alter the SPM landscape [45]. Therefore, future research is warranted to assess whether the broader application of targeted therapies might alter the SPM spectrum of solid and hematological cancers in patients with CLL. Also, future research with more detailed patient-level data on CLL-specific characteristics, the entire treatment landscape, and non-treatment-related exposures that may increase the risk of cancer development (e.g., tobacco use and UV exposure) should adopt multi-state modeling to explore sequences of these exposures on SPM development [46].

The strength of our study is the use of population-based data from a comprehensive, long-running, and well-established cancer registry, enabling us to accurately quantify the risk of developing an SPM over a 30-year period post-diagnosis. Limitations concern the lack of information on the exposure to well-known carcinogens and the exact therapeutic regimens during the overall course of the disease.

Collectively, identifying and managing SPMs is an essential part of the longevity of patients with cancer, especially in diseases with therapeutic advances that contribute to a noticeable improvement in survival, such as in CLL. Our findings can be used in shared decision-making about appropriate surveillance activities and interventions to counteract the increased morbidity and excess mortality associated with SPMs. The current study serves as a benchmark to assess how the spectrum of SPMs may alter with a broacher application of targeted therapies.

## Supplementary information

Supplemental Information

## Data Availability

The data that support the findings of this study are available via The Netherlands Comprehensive Cancer Organisation. These data are not publicly available, and restrictions apply to the availability of the data used for the current study. However, these data are available upon reasonable request and with permission of The Netherlands Comprehensive Cancer Organisation.
