# Supplementary material for: Risk of second primary malignancies in patients with chronic lymphocytic leukemia: a population-based study in the Netherlands, 1989-2019
Source: Blood Cancer J. 2023 Jan 13;13(1):15. doi: 10.1038/s41408-023-00784-z (PMC9837130; doi:10.1038/s41408-023-00784-z)

ONLINE APPENDIX

Title

Risk of second primary malignancies in patients with chronic lymphocytic leukemia: a population-based study in the Netherlands, 1989-2019

Short title

Secondary primary malignancies in CLL

Authors and affiliation

Lina van der Straten,[1-3] Mark-David Levin,[2] Manette A.W. Dinnessen,[1,4] Otto Visser,[5] Eduardus F.M. Posthuma,[6,7] Jeanette K. Doorduijn,[8] Anton W. Langerak,[3] Arnon P. Kater,[4] Avinash G. Dinmohamed[1,4,9,10]

[1]Department of Research and Development, Netherlands Comprehensive Cancer Organisation (IKNL), Utrecht, The Netherlands; [2]Department of Internal Medicine, Albert Schweitzer Hospital, Dordrecht, The Netherlands; [3]Department of Immunology, Erasmus MC, Rotterdam; [4]Amsterdam UMC, University of Amsterdam, Department of Hematology, Cancer Center Amsterdam, Lymphoma and Myeloma Center Amsterdam, Amsterdam, The Netherlands; [5]Department of Registration, Netherlands Comprehensive Cancer Organisation (IKNL), Utrecht, The Netherlands; [6]Department of Internal Medicine, Reinier The Graaf Hospital, Delft, The Netherlands; [7]Department of Hematology, Leiden University Medical Center, Leiden, The Netherlands; [8]Erasmus MC Cancer Institute, Department of Hematology, University Medical Center Rotterdam, Rotterdam, The Netherlands; [9]Erasmus MC, Department of Public Health, University Medical Center Rotterdam, Rotterdam, The Netherlands; [10]Amsterdam UMC, Vrije Universiteit Amsterdam, Department of Hematology, Cancer Center Amsterdam, Amsterdam, The Netherlands

Supplemental Table 1

Supplemental Table 1. Definitions of the subtypes of secondary primary malignancies with corresponding topography and morphology codes as per the International Classification of Diseases for Oncology

SPM category	Tumor subtype	Topography	Morphology
Oral cavity and pharynx	Tongue carcinoma	C02	8000-8576, 8940-8941, 8980, 8982, 9110, 9990
	Gum carcinoma	C03	8000-8576, 8940-8941, 8980, 8982, 9110, 9990
	Floor of the mouth carcinoma	C04	8000-8576, 8940-8941, 8980, 8982, 9110, 9990
	Palate carcinoma	C05.0, C05.8-9	8000-8576, 8940-8941, 8980, 8982, 9110, 9990
	Oral cavity, other/NOS	C00.3-5, C06	8000-8576, 8940-8941, 8980, 8982, 9110, 9990
	Oropharynx carcinoma	C01, C05.1-2, C09, C10.0, C10.2-4, C10.8-9	8000--8576, 8940-8941, 8980, 8982, 9110, 9990
	Nasopharynx carcinoma	C11	8000-8576, 8940-8941, 8980, 8982, 9110, 9990
	Hypopharynx carcinoma	C12-13	8000-8576, 8940-8941, 8980, 8982, 9110, 9990
	Mouth and pharynx, other/NOS	C14.0, C14.2, C14.9	8000-8576, 8940-8941, 8980, 8982, 9110, 9990
	Parotid gland carcinoma	C07	8000-8576, 8940-8941, 8980, 8982, 9110, 9990
	Other salivary glands	C08	8000-8576, 8940-8941, 8980, 8982, 9110, 9990
Esophagus	Cervical oesophageal carcinoma	C15.0	8000-8149, 8154, 8158-8230, 8243-8246, 8250-8576, 8980, 8982, 9110, 9990
	Oesophageal carcinoma (excl. cervical)	C15.1-9	8000-8149, 8154, 8158-8230, 8243-8246, 8250-8576, 8980, 8982, 9110, 9990
Stomach	Invasive cardia carcinoma	C16.0	8000-8149, 8154, 8158-8230, 8243-8246, 8250-8576, 8980, 8982, 9110, 9990
	Invasive stomach carcinoma	C16.1-9	8000-8149, 8154, 8158-8230, 8243-8246, 8250-8576, 8980, 8982, 9110, 9990
Colon and rectum	Colon carcinoma	C18.0, C18.2-9	8000-8231, 8243-8246, 8250-8576, 8980, 8982, 9110, 9990
	Appendix carcinoma	C18.1	8000-8231, 8243-8246, 8250-8576, 8980, 8982, 9110, 9990
	Rectum carcinoma	C20	8000-8231, 8243-8246, 8250-8576, 8980, 8982, 9110, 9990
	Rectosigmoid carcinoma	C19	8000-8231, 8243-8246, 8250-8576, 8980, 8982, 9110, 9990
Pancreas	Exocrine pancreatic carcinoma	C25	8012, 8014-8040, 8045-8148, 8154, 8160-8231, 8243-8245, 8250-8573, 8575-8576, 8980, 8982
	Pancreatic carcinoma	C25.0-3, C25.5-9	8000-8011, 9990

Supplemental Table 1 (continued)

SPM category	Tumor subtype	Topography	Morphology
Pancreas	Exocrine pancreatic carcinoma	C25	8012, 8014-8040, 8045-8148, 8154, 8160-8231, 8243-8245, 8250-8573, 8575-8576, 8980, 8982
	Pancreatic carcinoma	C25.0-3, C25.5-9	8000-8011, 9990
Larynx	Supraglottic carcinoma	C32.1, C10.1	8000-8576, 8940-8941, 8980, 8982, 9110, 9990
	Glottic carcinoma	C32.0	8000-8576, 8940-8941, 8980, 8982, 9110, 9990
	Subglottic carcinoma	C32.2	8000-8576, 8940-8941, 8980, 8982, 9110, 9990
	Other/unspecified laryngeal carcinoma	C32.8-9	8000-8576, 8940-8941, 8980, 8982, 9110, 9990
Lung and bronchus	Non-small-cell lung carcinoma	C34	8010-8020, 8022-8035, 8046-8230, 8243-8246, 8250-8576, 8972, 8980-8982, 9110
	Small-cell lung carcinoma	C34	8002, 8021, 8041-8045
	Carcinoid of the lung	C34	8240-8242, 8248-8249
	Other/unspecified lung cancer	C34	8000-8001, 8003-8005, 9990, 8720-8790
	Pleuropulmonal blastoma	C34	8973
Melanoma skin	Melanoma of the skin and lip	C00.0, C00.1, C44	8720-8790
	Melanoma of the vulva	C51	8720-8790
	Melanoma of the penis	C60	8720-8790
	Melanoma of the scrotum	C63.2	8720-8790
	Melanoma of a primary unknown site	C80	8720-8790
Squamous cell skin	Squamous cell carcinoma of the eyelid	C44.1	8000-8012, 8014-8035, 8050-8084, 8560, 8575, 8980, 9990
	Squamous cell carcinoma and other skin sites	C44.5-9, C63.2	8000-8012, 8014-8035, 8050-8084, 8560, 8575, 8980, 9990
	Squamous cell carcinoma head and neck	C44.0, C44.2-4	8000-8012, 8014-8035, 8050-8084, 8560, 8575, 8980, 9990
Breast	Invasive breast cancer carcinoma	C50	8000-8576, 8980, 8982, 8983, 9110, 9990
Endometrium	Endometrium carcinoma	C54-55	8000-8576, 8950-8951, 8980-8982, 9110, 9990

Supplemental Table 1 (continued)

SPM category	Tumor subtype	Topography	Morphology
Ovary	Borderline ovarian carcinoma	C56, C48.1-2, C57	8000-8576, 9000-9015, 9110
	Borderline ovarian carcinoma	C56	8460
	Borderline ovarian carcinoma	C56	8442, 8451, 8462, 8463, 8472, 8473
	Non-epithelial ovarian tumors borderline	C56, C48.1-2, C57	8590-8670, 9060-9105
	Non-invasive tuba carcinoma	C57.0	8000-8576, 8930-8934, 8950-8951, 8980, 8982, 9000-9015, 9110, 9990
	Epithelial ovarian carcinoma	C56	8000-8239, 8250-8441, 8450, 8452-8461, 8470-8471, 8474, 8480-8576, 8930-8934, 8950-8951, 8980, 8982, 9000-9015, 9110, 9990
	Extra-ovarian carcinoma	C48.1-2	8000, 8010-8149, 8154, 8158-8231, 8250-8576, 8950-8951, 8980, 8982, 9110
	Non-epithelial ovarian cancer	C56	8240-8249, 8590-8670, 9060-9105, 8800-8831, 8840-8850, 8852-8921, 8935, 8960-8973, 8990-8991, 9040-9044, 9120-9133, 9150-9261, 9364, 9380-9514, 9530-9581
Prostate	Prostate carcinoma	C61	8000-8576, 8980, 8982, 9110, 9990
Urinary bladder	Muscle-invasive bladder carcinoma	C67.0-6, C67.8-9	8000-8576, 8980, 8982, 9110, 9990
Kidney and renal pelvis	Kidney carcinoma	C64	8000-8576, 8980, 8982, 9110, 9990
	Carcinoma of the renal pelvis carcinoma	C65	8000-8576, 8980, 8982, 9110, 9990
Brain	Malign brain tumors, neuroepithelial	C71, C72.2-3, C75.1, C75.3	9380-9420, 9422-9514
	Malign brain tumors, unspecified	C71, C72.2-3	8000-8005, 9990
Thyroid	Papillary and follicular carcinoma of the thyroid gland	C73	8010, 8046, 8140, 8201, 8260, 8290, 8310, 8330, 8331, 8332, 8335, 8337, 8339, 8340, 8341, 8342, 8343, 8344, 8350, 8430, 8450, 8481, 8504, 8560, 8570
	Medullary carcinoma of the thyroid gland	C73	8240, 8246, 8345-8347, 8510-8511
	Anaplastic carcinoma of the thyroid gland	C73	8012, 8020, 8021, 8022, 8030, 8031, 8032, 8033, 8035, 8041, 8980
	Squamous cell carcinoma of the thyroid gland	C73	8051-8084
	Other/unspecified carcinoma of the thyroid gland	C73	8000-8005, 9990, 8588, 8589

Supplemental Table 1 (continued)

Soft-tissue sarcoma	Soft tissue sarcoma of the head and neck	C44.0-4, C47.0, C49.0, C00-14, C30-32, C73	8710-8711, 8714, 8800-8831, 8840-8850, 8852-8921, 8935, 8963-8964, 8990-8991, 9040-9045, 9137, 9150-9170, 9180-9261, 9364-9365, 9540-9581
	Soft tissue sarcoma of the head and neck	C47.0, C49.0	8000-8005, 8980-8982, 9990
	Soft tissue sarcoma of the head and neck	C44.0-4, C49.0, C00-14, C30-32, C73	9120-9133
	Soft tissue sarcoma of the extremities	C44.6-7, C47.1-2, C49.1-2	8710-8711, 8714, 8800-8831, 8840-8850, 8852-8921, 8935, 8963-8964, 8990-8991, 9040-9044, 9137, 9150-9170, 9180-9261, 9364-9365, 9540-9581
	Soft tissue sarcoma of the extremities	C47.1-2, C49.1-2	8000-8005, 8980-8982, 9990
	Soft tissue sarcoma of the extremities	C44.6-7, C49.1-2	9120-9133
	Soft tissue sarcoma of the thorax	C47.3, C49.3, C38.1-3, C38.8	8000-8005, 8710-8711, 8714, 8800-8831, 8840-8850, 8852-8921, 8935, 8963-8964, 8980-8982, 8990-8991, 9040-9044, 9137, 9150-9170, 9180-9261, 9364-9365, 9540-9581, 9990
	Soft tissue sarcoma of the thorax	C38.4	8710-8711, 8714, 8800-8831, 8840-8850, 8852-8921, 8935, 8963-8964, 8980-8982, 8990-8991, 9040-9044, 9120-9133, 9137, 9150-9170, 9180-9261, 9364-9365, 9540-9581
	Soft tissue sarcoma of the thorax	C37, C39	8710-8711, 8714, 8800-8831, 8840-8850, 8852-8921, 8935, 8963-8964, 8990-8991, 9040-9044, 9120-9133, 9137, 9150-9170, 9180-9261, 9364-9365, 9540-9581
	Soft tissue sarcoma of the thorax	C44.5, C49.3, C38.1-3, C38.8	9120-9133
	Soft tissue sarcoma of the thorax	C38.0	8000, 8710-8711, 8714, 8800-8831, 8840-8850, 8852-8921, 8935, 8963-8964, 8990-8991, 9040-9044, 9120-9133, 9137, 9150-9170, 9180-9261, 9364-9365, 9540-9581, 9990
	Sarcoma of the lung and trachea	C33-C34	8710-8711, 8714, 8800-8831, 8840-8850, 8852-8921, 8935, 8963-8964, 8990-8991, 9040-9044, 9120-9133, 9137, 9150-9170, 9180-9261, 9364-9365, 9540-9581
	Soft tissue sarcoma of the abdomen, retroperitoneum, and pelvis	C47.4-5, C49.4-5, C48.0	8000-8005, 8710-8711, 8714, 8800-8831, 8840-8850, 8852-8921, 8935, 8963-8964, 8980-8982, 8990-8991, 9040-9044, 9137, 9150-9170, 9180-9261, 9364-9365, 9540-9581, 9990

Supplemental Table 1 (continued)

Soft-tissue sarcoma	Soft tissue sarcoma of the abdomen, retroperitoneum, and pelvis	C74	8710-8711, 8714, 8800-8831, 8840-8850, 8852-8921, 8935, 8963-8964, 8980-8982, 8990-8991, 9040-9044, 9137, 9150-9170, 9180-9261, 9364-9365, 9540-9581
	Soft tissue sarcoma of the abdomen, retroperitoneum, and pelvis	C42.2, C49.4-5, C48.0, C74	9120-9133
	Soft tissue sarcoma - other/NOS	C44.5, C44.8-9, C47.6-9, C49.6-9, C80	8000-8005, 8710-8711, 8714, 8800-8831, 8840-8850, 8852-8921, 8935, 8963-8964, 8980-8982, 8990-8991, 9040-9044, 9120-9133, 9137, 9150-9170, 9180-9261, 9364-9365, 9540-9581, 9990
Non-Hodgkin lymphoma	Follicular lymphoma	all	9690-9696, 9615, 9623, 9633
	Lymphoplasmacytic lymphoma / Waldenström macroglobulinemia	all	9761, 97611
	Indolent NHL, other/NOS	all	9689, 9711, 9764
	Hairy cell leukemia	all	9940, 9941
	B-PLL	all	9825, 9832, 9833, 9631
	Mantle cell lymphoma	all	9673, 9621, 9672
	Burkitt lymphoma/leukemia	all	9687, 9826
Multiple myeloma	Multiple myeloma	all	9730, 9732
Acute myeloid leukemia	AML with specific cytogenetic abnormalities	all	9865-9866, 9869, 9871, 9877-9879, 9896-9897, 9911, 9912
	AML with myelodysplasia-related changes	all	9895, 9984
	Therapy-related myeloid neoplasms	all	9920, 9987
	AML, other/NOS	all	9840-9841, 9861, 9864, 9867, 9870, 9872-9874, 9890-9891, 9910, 9931-9932
	Myeloid sarcoma	all	9930
	Myeloid leukemia associated with Down syndrome	all	9898
	Blastic plasmacytoid dendritic cell neoplasm	all	9727

Supplemental Table 1 (continued)

Myeloproliferative neoplasms*	Chronic myeloid leukemia	all	9863, 9875
	Mast cell tumors	all	9740-9742, 9900
	Myelofibrosis	all	9961
	Myeloid and lymphoid neoplasms with eosinophilia and abnormalities of PDGFRA, PDGFRB or FGFR1	all	9965-9967, 9963-9964, 9968, 9880
	Myeloproliferative neoplasms, other/NOS	all	9842, 9950, 9960, 9962
Myelodysplastic syndrome*	Myelodysplastic syndrome	all	9980, 9982-9983, 9985-9986, 9989, 9991-9993
Primary site unknown	Other/unspecified sites	C76, C39	8000-8576, 8980, 8982, 9110, 9990
	Primary site unknown	C80	8000-8148, 8154, 8160-8231, 8243-8246, 8250-8576, 8980-8982, 9110, 9990

*Information on myeloproliferative neoplasms and myelodysplastic syndromes is available as of 2001 since the WHO first recognized these entities in the WHO 2001 classification as malignant disorders. Abbreviations: AML, acute myeloid leukemia; NHL, non-Hodgkin lymphoma; NOS, not otherwise specified; PLL, prolymphocytic leukemia; WHO, World Health Organization.

Supplemental Table 2

Supplemental Table 2. Standardized incidence ratios and absolute excess risk of all SPMs and SPM subtypes according to the calendar period

	1989-1995				1995-2002				2003-2009				2010-2019			
**Type of SPM**	**Obs**	**SIR (95%CI)**		**AER**	**Obs**	**SIR (95%CI)**		**AER**	**Obs**	**SIR (95%CI)**		**AER**	**Obs**	**SIR (95%CI)**		**AER**
All sites	711	**1.65**	**(1.53-1.77)**	119.23	1,007	**1.60**	**(1.51-1.71)**	114.59	1,507	**1.71**	**(1.62-1.80)**	143.08	1,145	**1.56**	**(1.47-1.66)**	116.72
Any solid cancer	687	**1.66**	**(1.54-1.79)**	116.01	985	**1.65**	**(1.54-1.75)**	116.41	1,453	**1.73**	**(1.64-1.82)**	139	1,099	**1.57**	**(1.48-1.66)**	111.96
Oral cavity or pharynx	4	0.56	(0.15-1.43)	-1.21	15	1.33	(0.75-2.20)	1.01	23	1.39	(0.88-2.09)	1.31	12	0.88	(0.45-1.54)	-0.42
Gastrointestinal tract																
Esophagus	10	1.10	(0.53-2.02)	0.33	11	0.72	(0.36-1.28)	-1.17	29	1.24	(0.83-1.78)	1.12	17	0.87	(0.51-1.40)	-0.64
Stomach	25	1.43	(0.92-2.11)	2.86	27	1.40	(0.92-2.03)	2.05	24	1.11	(0.71-1.65)	0.48	11	0.78	(0.39-1.40)	-0.79
Colon and rectum	85	1.18	(0.94-1.46)	4.97	123	1.15	(0.96-1.38)	4.47	205	**1.37**	**(1.19-1.58)**	11.43	120	1.06	(0.88-1.27)	1.73
Pancreas	24	**1.97**	**(1.26-2.93)**	4.51	15	0.87	(0.49-1.44)	-0.59	19	0.77	(0.46-1.20)	-1.16	26	1.30	(0.85-1.90)	1.54
Lower respiratory system																
Larynx	5	1.05	(0.34-2.44)	0.08	11	1.74	(0.87-3.11)	1.25	12	1.52	(0.78-2.65)	0.82	2	0.35	(0.04-1.25)	-0.98
Lung or bronchus	111	**1.48**	**(1.22-1.78)**	13.76	149	**1.45**	**(1.23-1.70)**	12.46	186	**1.35**	**(1.16-1.56)**	9.77	127	1.19	(0.99-1.42)	5.30
Skin																
Melanoma	38	**3.79**	**(2.68-5.20)**	10.75	53	**2.61**	**(1.96-3.42)**	8.84	100	**2.76**	**(2.24-3.35)**	12.97	87	**2.50**	**(2.00-3.08)**	13.56
Squamous cell	196	**5.71**	**(4.94-6.56)**	63.69	339	**5.46**	**(4.90-6.07)**	77.08	532	**4.91**	**(4.50-5.34)**	88.81	420	**4.04**	**(3.66-4.44)**	83.84
Breast	43	0.96	(0.70-1.30)	-0.62	67	1.10	(0.86-1.40)	1.73	91	1.16	(0.94-1.43)	2.60	58	0.97	(0.74-1.25)	-0.47
Female genital organ																
Endometrium	5	0.65	(0.21-1.51)	-1.04	16	1.54	(0.88-2.51)	1.52	10	0.75	(0.36-1.38)	-0.67	12	1.21	(0.63-2.12)	0.55
Ovary	4	0.63	(0.17-1.60)	-0.91	9	1.22	(0.56-2.31)	0.43	7	0.79	(0.32-1.62)	-0.38	8	1.27	(0.55-2.50)	0.44
Male genital organ																
Prostate	75	1.12	(0.88-1.41)	3.16	112	1.08	(0.89-1.30)	2.33	159	1.08	(0.92-1.26)	2.35	130	1.12	(0.93-1.33)	3.60
Urinary tract																
Urinary bladder or renal pelvis	26	1.13	(0.71-1.66)	1.17	34	1.05	(0.72-1.46)	0.41	57	1.31	(0.99-1.70)	2.74	35	1.08	(0.75-1.50)	0.63
Kidney	18	**1.71**	**(1.01-2.70)**	2.85	26	**1.62**	**(1.06-2.37)**	2.68	49	**2.09**	**(1.55-2.76)**	5.18	27	1.41	(0.93-2.05)	2.05
Brain	9	2.17	(0.99-4.12)	1.85	7	1.10	(0.44-2.26)	0.17	14	1.56	(0.85-2.61)	1.01	6	0.84	(0.31-1.83)	-0.29
Thyroid gland	1	0.78	(0.02-4.35)	-0.11	2	1.04	(0.13-3.77)	0.02	5	1.76	(0.57-4.12)	0.44	10	**4.08**	**(1.96-7.51)**	1.95

Supplemental Table 2 (continued)

Soft-tissue sarcoma	8	**2.88**	**(1.20-5.68)**	1.99	8	**2.08**	**(0.90-4.10)**	1.12	17	**3.20**	**(1.86-5.12)**	2.36	6	1.37	(0.50-2.99)	0.42
Primary site unknown	36	**1.76**	**(1.23-2.44)**	5.93	42	**1.82**	**(1.31-2.46)**	5.08	37	**1.69**	**(1.19-2.34)**	3.06	14	1.16	(0.63-1.94)	0.50
Blood, bone marrow, or lymphatic system																
Any hematological cancer	31	1.41	(0.96-2.00)	3.45	37	1.38	(1.38-0.71)	0.02	85	**1.55**	**(1.24-1.91)**	6.11	71	**1.63**	**1.27-2.05**	7.13
Non-Hodgkin lymphoma	16	**2.33**	**(1.33-3.78)**	3.49	11	1.10	(0.55-1.97)	0.27	22	1.54	(0.96-2.33)	1.55	10	0.83	0.40-1.53	-0.53
Multiple myeloma	3	0.46	(0.10-1.36)	-1.32	5	0.54	(0.18-1.27)	-1.13	12	0.93	(0.48-1.62)	-0.19	20	1.56	0.99-3.03	2.54
Acute myeloid leukemia	6	1.76	(0.65-3.83)	0.99	10	2.02	(0.97-3.72)	1.36	25	**3.72**	**(2.41-5.49)**	3.69	15	**2.85**	**1.59-4.69**	2.51
Myeloproliferative neoplasm	1	0.43	(0.01-2.42)	-0.50	2	0.36	(0.04-1.31)	-0.94	6	0.62	(0.23-1.35)	-0.73	8	0.98	0.42-1.94	-0.04
Myelodysplastic syndromes	2	0.88	(0.11-3.17)	-0.11	2	0.31	(0.04-1.13)	-1.17	14	1.37	(0.75-2.31)	0.77	17	**2.35**	**1.37-3.77**	2.53

Statically significant SIRs are depicted in bold.
Abbreviations: AER, absolute excess risk; CI, confidence interval; SIR, absolute excess risk; and Obs, observed.

Supplemental Table S3

Supplemental Table S3. The spectrum of SPM development according per treatment category, 2014-2019

Treatment	n per treatment category	n of developed SPMs	SPM category†	n per SPM category
FCR	207	23	Squamous cell skin	9
			Lung and bronchus	6
			Acute myeloid leukemia	2
			Prostate	2
			Colon and rectum	1
			Breast	1
			Melanoma skin	1
			Urinary bladder and renal pelvis	1
R or O-Clb	199	24	Squamous cell skin	18
			Colon and rectum	3
			Lung and bronchus	3
			Melanoma skin	2
			Oral cavity and pharynx	1
			Stomach	1
			Prostate	1
			Urinary bladder and renal pelvis	1
			Kidney	1
			Multiple myeloma	1
			Myelodysplastic syndrome	1
			Primary site unknown	1
Clb monotherapy	79	3	Squamous cell skin	2
			Colon and rectum	1
R-CVP	75	10	Squamous cell skin	2
			Lung and bronchus	2
			Acute myeloid leukemia	1
			Breast	1
			Melanoma skin	1
			Myelodysplastic syndrome	1
			Primary site unknown	1
			Prostate	1
BR	70	11	Squamous cell skin	8
			Prostate	2
			Colon and rectum	1
Other*	67	6	Squamous cell skin	3
			Acute myeloid leukemia	1
			Esophagus	1
			Myelodysplastic syndrome	1
Venetoclax	34	1	Squamous cell skin	1
Ibrutinib	27	1	Prostate	1

*The other treatment category consisted of rituximab plus prednisone or methotrexate (n=50), R-CHOP (n=11), fludarabine monotherapy (n=3), FC (n=1), bendamustine monotherapy (n=1) and R-idelalisib (n=1).

Abbreviations: BR, bendamustine and rituximab; Clb, chlorambucil, FC, fludarabine and cyclophosphamide; FCR, fludarabine, cyclophosphamide and rituximab; n, number; R-CHOP, rituximab, cyclophosphamide, doxorubicin, vincristine and prednisone; R-CVP, rituximab, cyclophosphamide, vincristine, and prednisone; R or O-Clb, rituximab or obinutuzumab with chlorambucil; ICD-code, International Classification of Diseases for Oncology code.

Supplemental Figure legends

Supplemental Figure 1. Cumulative incidence (one minus Kaplan-Meier estimate) of subtypes of second primary malignancies among patients with chronic lymphocytic leukemia and the general Dutch population. Solid blue lines represent the observed incidence in patients with chronic lymphocytic leukemia, and the dashed pink lines the expected incidence in the general population. Please note the alternative Y-scale for squamous cell caricomonas of the skin.

Supplemental Figure 2. Standardized incidence ratios (A) and absolute excess risk (B) of all second primary malignancies among patients with chronic lymphocytic leukemia according to the latency time. The standardized incidence ratio (SIRs) compares the observed cancer incidence among the studied cohort with the expected cancer incidence in an age-, sex- and period-matched group from the general population. The absolute excess risk (AER) represents the additional cancer risk among patients with chronic lymphocytic leukemia compared to the general population. The overall SIR and AER are depicted as a diamond, and the individual SIR and AER circles are scaled according to their magnitude. The statistically significant SIRs and AER are presented in bold, and the vertical lines depict the 95% confidence interval.

Supplemental Figure 3. Standardized incidence ratios (A) and absolute excess risk (B) of all site second primary malignancies among patients with chronic lymphocytic leukemia according to the age categories. The standardized incidence ratio (SIRs) compares the observed cancer incidence among the studied cohort with the expected cancer incidence in an age-, sex- and period-matched group from the general population. The absolute excess risk (AER) represents the additional cancer risk among patients with chronic lymphocytic leukemia compared to the general population. The overall SIR and AER are depicted as a diamond, and the individual SIR and AER circles are scaled according to their magnitude. The statistically significant SIRs and AER are presented in bold, and the vertical lines depict the 95% confidence interval.

Supplemental Figure 1

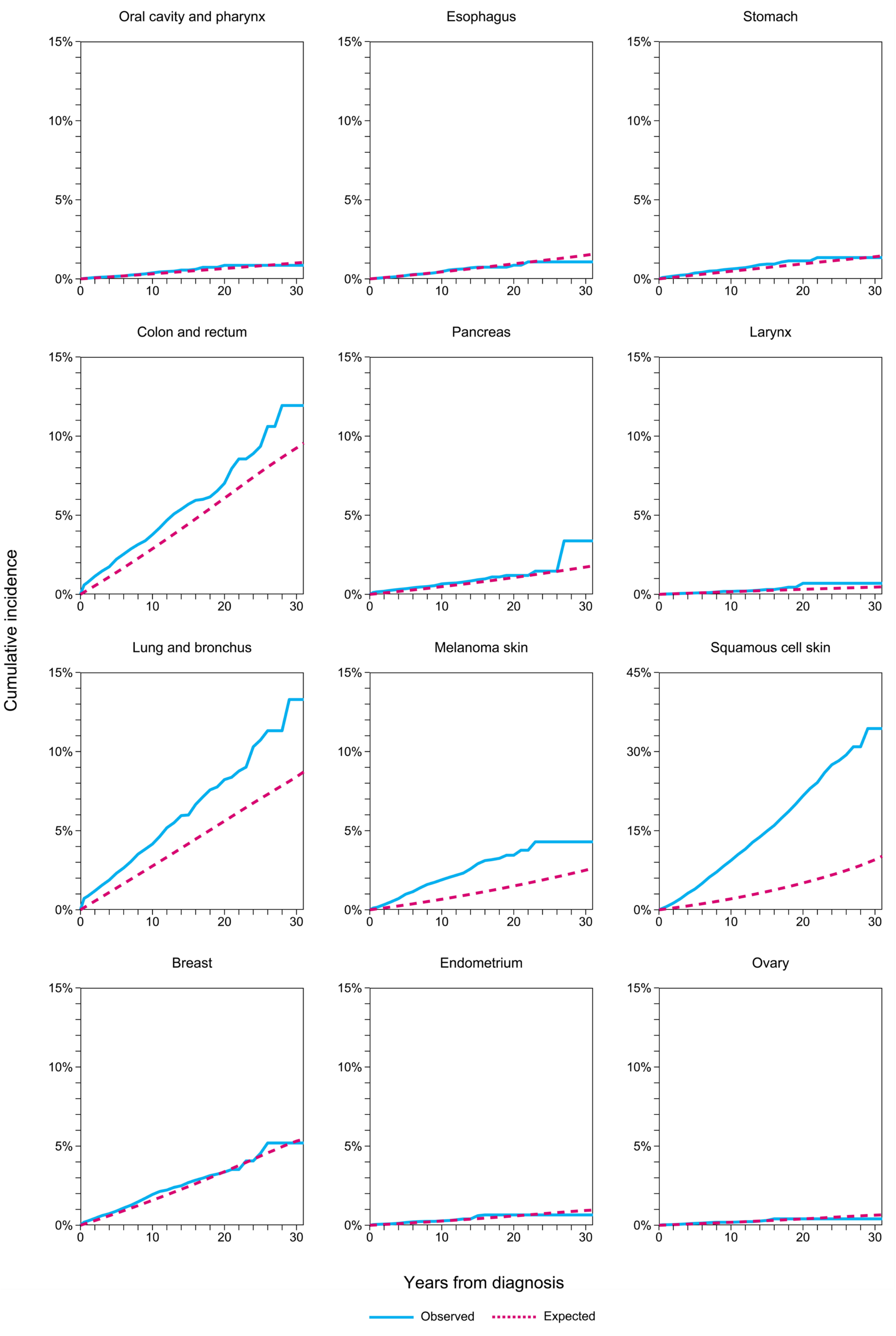

Supplemental Figure 1 (continues)

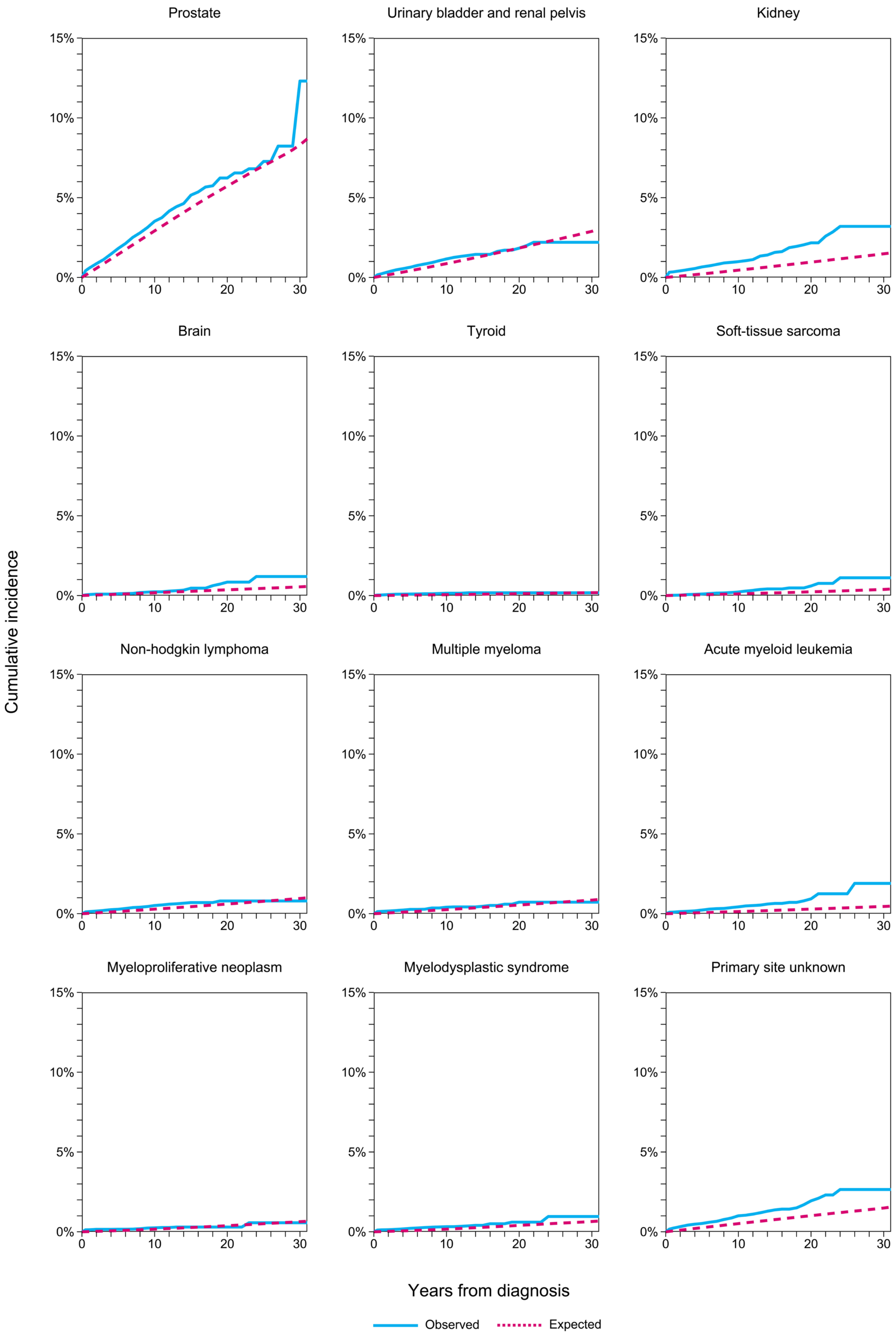

Supplemental Figure 2

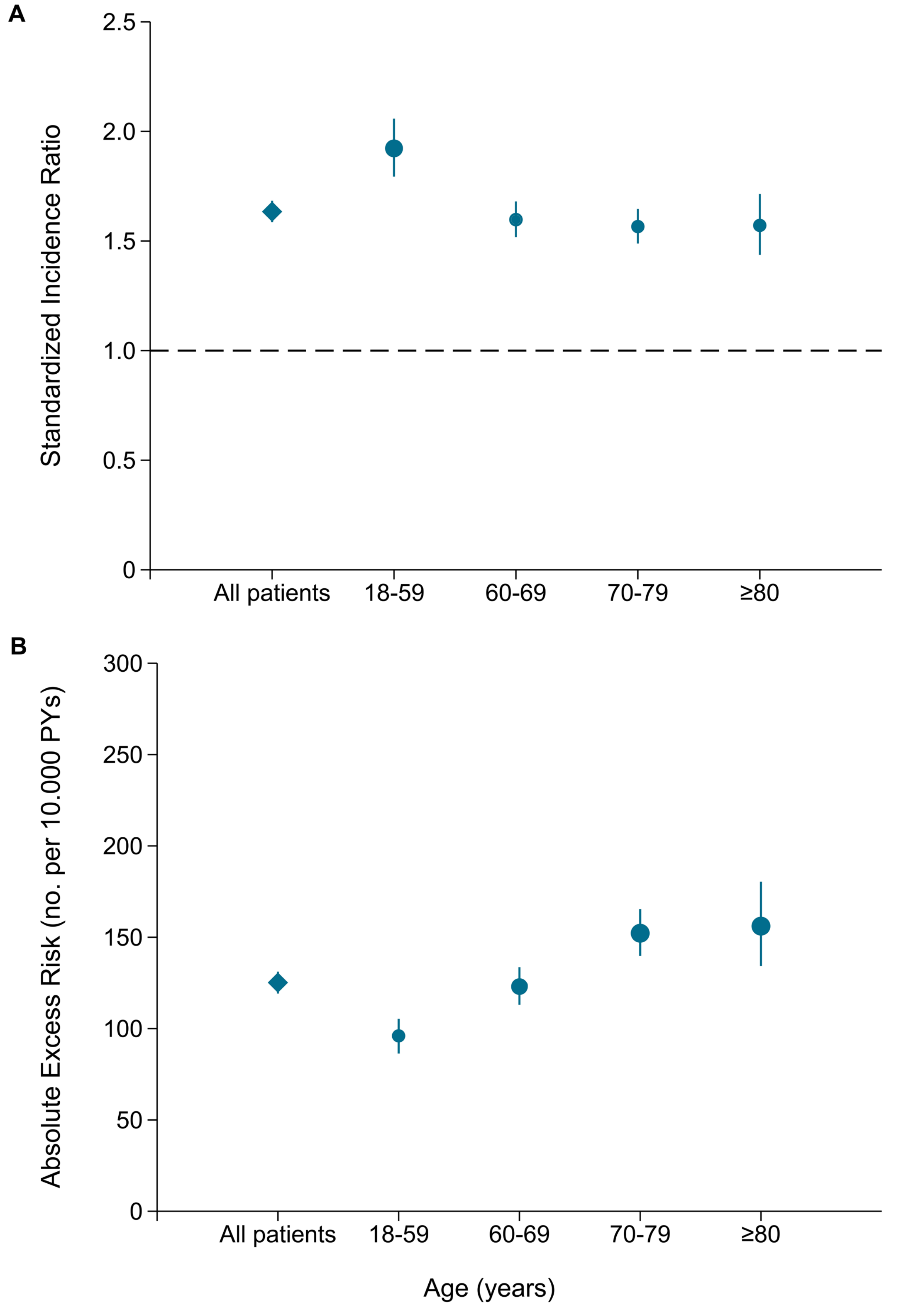

Supplemental Figure 3

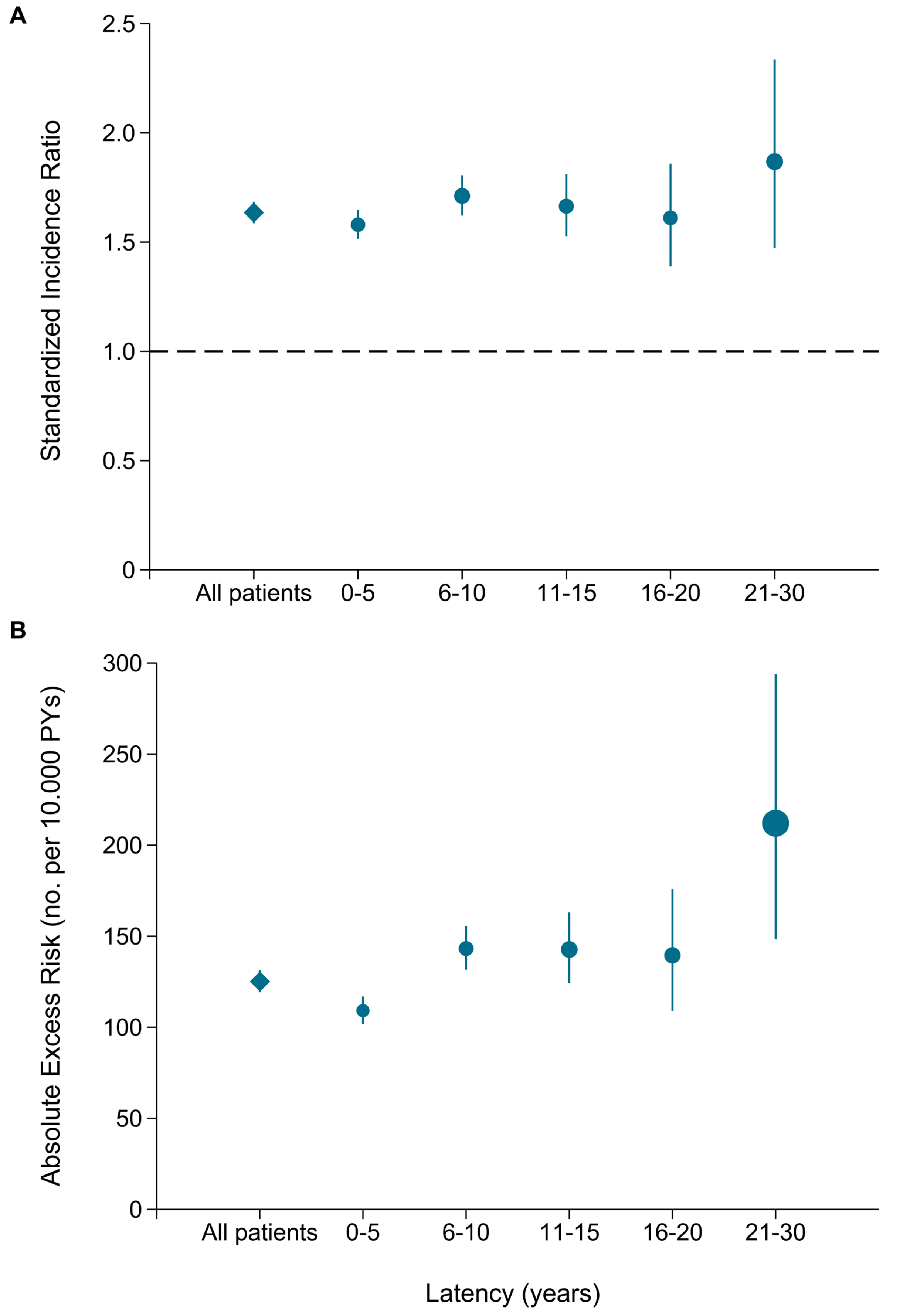

Supplement: Supplementary file 1 — Supplemental Information [file 41408_2023_784_MOESM1_ESM.pdf]
